# Management of Respiratory Illness in a Pediatric Patient With Chromosome 16p13.3 Microduplication Syndrome and Potential Seizure Risk

**DOI:** 10.7759/cureus.93668

**Published:** 2025-10-01

**Authors:** Mikaela A Lee, Travis Smith, Katarzyna Madejczyk

**Affiliations:** 1 Pediatric Emergency Department, AdventHealth Daytona Beach, Daytona Beach, USA; 2 Clinical Curriculum Integration &amp; Assessment, Lake Erie College of Osteopathic Medicine (LECOM), Bradenton, USA

**Keywords:** chromosome, genetics, microduplication, pediatrics, pediatric seizure, prevention, respiratory, seizure

## Abstract

This case report outlines the management of a 16-month-old girl with chromosome 16p13.3 microduplication syndrome who presented with an acute respiratory illness. For signs and symptoms of croup, pneumonia, and otitis media, this patient required multiple administrations of racemic epinephrine, steroids, and albuterol to reduce inflammation and alleviate respiratory distress. Children with underlying medical conditions and genetic syndromes are likely to have a more complicated clinical course in the setting of acute illnesses. Thus, these patients require more intensive interventions and prolonged monitoring than the standard protocols. In this case, the treatment regimen was carefully tailored, with special attention to minimizing the risk of seizure activity. Furthermore, this case highlights the need for further exploration of the interaction between respiratory illnesses and underlying seizure disorders. The patient’s presentation underscores the importance of neurological observation and a multidisciplinary approach to ensure the best possible outcome.

## Introduction

Chromosome 16p13.3 microduplication results from a partial duplication of the short arm of chromosome 16, which can lead to a wide range of features among affected individuals [1]. This rare genetic disorder is estimated to occur in fewer than one in a million people worldwide, with symptoms typically emerging in infants [1]. There are three inheritance patterns: spontaneous duplication (de novo), autosomal dominant inheritance, or mosaicism. Recent studies have found chromosome 16 to be one of the least stable regions in the human genome, making it a vulnerable site for recurrent microdeletions and duplications [2]. Common genes in this region were found to involve in brain and neurological function as well as immune system development. Although more commonly associated with Rubinstein-Taybi syndrome (RTS), the CREB Binding Protein (*CREBBP*) gene has been identified as the primary gene of the 16p13.3 microduplication phenotype, along with key genes Glutamate Ionotropic Receptor NMDA Type Subunit 2A (*GRIN2A*) and RNA Binding Fox-1 Homolog 1 (*RBFOX1*) [3]. All three genes are critical in neural development and can increase the risk of developing epilepsy. The most proximal gene to the duplication site is *RBFOX1*, which can predispose patients to seizures. While epilepsy is not a universal symptom in chromosome 16p13.3 microduplication, it is still present in a subset of individuals [4].

Chromosome 16p13.3 microduplication often presents with multiple clinical features affecting multiple systems, including respiratory, cardiac, musculoskeletal, and nervous systems [5]. Patients with chromosome 16p13.3 microduplication may be susceptible to respiratory illnesses not only due to upper airway defects or craniofacial anomalies but also due to a weak immune response [6]. The goal of this case report is to stress the significance of careful management of patients with chromosome 16p13.3 microduplication who are receiving antiepileptic drugs and to identify the need for increased respiratory support and monitoring. Given the rarity of the condition, it still remains underdiagnosed in the pediatric setting, highlighting the clinical implications of timely diagnosis and oversight.

## Case presentation

A 16-month-old female toddler with a known history of chromosome 16p13.3 microduplication and diffuse epileptic encephalopathy presented to the pediatric emergency department by her mother. She presented with worsening cough, change in voice during crying, noisy breathing, and shortness of breath for one day. She was afebrile and had no vomiting or diarrhea, but her mother was most concerned about the risk of seizures in the setting of her new respiratory illness. The patient had been following up routinely with her neurologist to monitor seizure activity, with her last seizure occurring 10 weeks ago. In addition, she was receiving outpatient antiepileptic therapy with clobazam, levetiracetam, and oxcarbazepine under regular neurological supervision.

**Figure 1:**
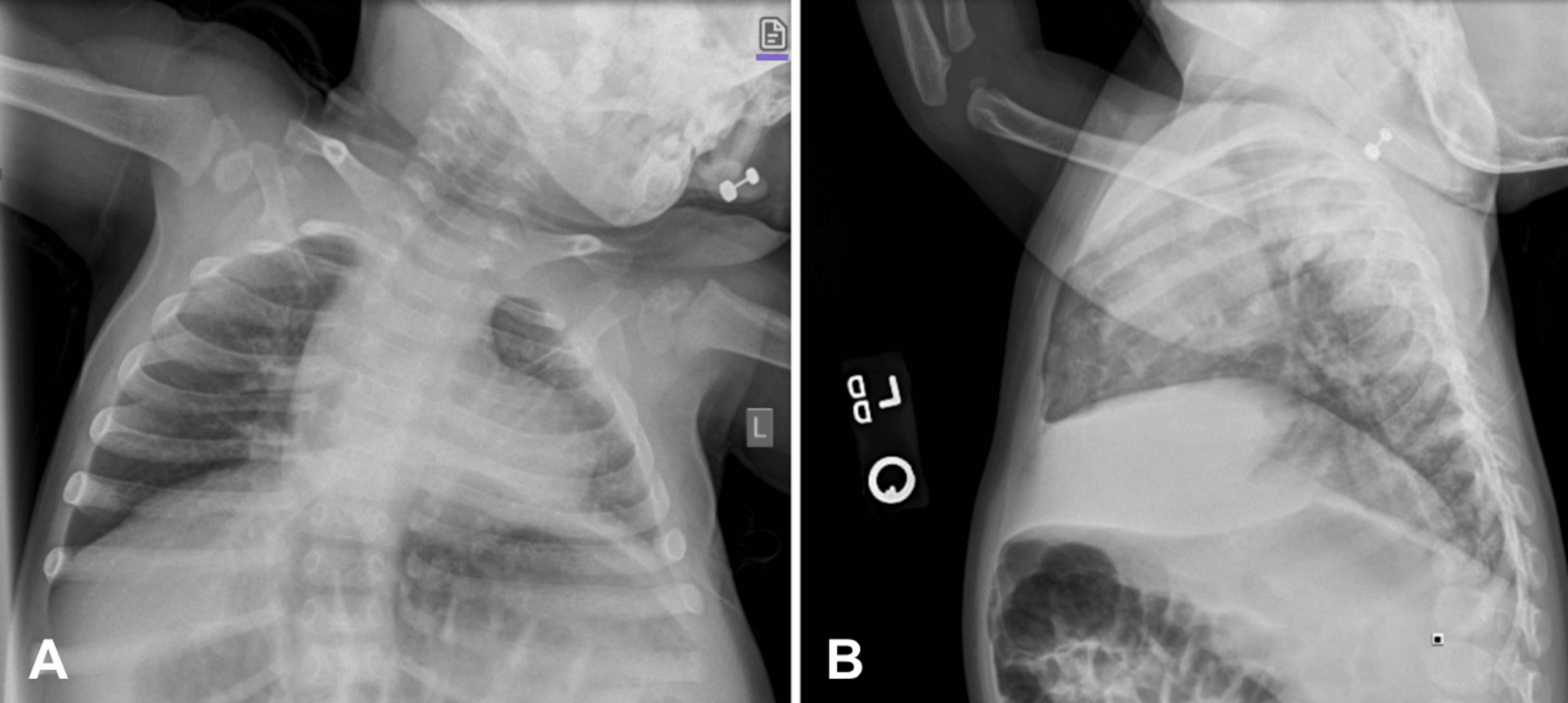
The patient’s chest X-rays with (A) AP and (B) lateral views on presentation were not indicative of signs of pneumonia, croup, or other respiratory disease. The lungs are clear, with no evidence of focal or patchy infiltrates, pleural effusion, or consolidations. No signs of airway narrowing. AP: anteroposterior.

Upon arrival at the emergency department, the patient was afebrile and hemodynamically stable (Table 1). She presented with symptoms of croup and was treated with oral dexamethasone and a racemic epinephrine nebulizer. A chest X-ray showed no obvious infiltrates or steeple sign (Figure 1). A respiratory panel was positive for parainfluenza virus 4 and mycoplasma pneumonia. Due to the continuation of stridor, congestion with pooled secretions, and wheezing, the patient was admitted for observation of worsening respiratory disease. 

**Table 1:** The patient’s vital signs on presentation demonstrated hemodynamic stability.

Vital signs	Value
Temperature (℉)	98.6
Heart rate (beats per minute)	117
Respiratory rate (breaths per minute)	26
Systolic/diastolic blood pressure (mmHg)	113/71

A follow-up chest radiograph was obtained to further evaluate for lobar pneumonia; however, findings were more consistent with viral bronchiolitis. The patient’s oxygen saturation was consistently between 96% and 100% on room air. She was given a 0.5 mg budesonide nebulizer twice a day for 14 days for parainfluenza infection, with nasal saline and suctioning every four hours as needed. A five-day course of systemic corticosteroids (e.g., oral prednisone) was administered, and albuterol was prescribed on an as-needed basis for bronchospasm. For the mycoplasma infection, she was given oral azithromycin.

On hospital day 2, a physical examination revealed a bulging tympanic membrane with mucopurulent discharge, consistent with acute otitis media. Intramuscular ceftriaxone was administered to provide broad-spectrum coverage for both otitis media and potential bacterial superinfection of the respiratory tract. The patient’s respiratory status appeared stable. Due to persistent nasal secretions and wheezing, she benefited from continuous suctioning and albuterol throughout the day. The patient demonstrated clinical improvement, with reduced wheezing, congestion, and improved airway clearance.

**Figure 2:**
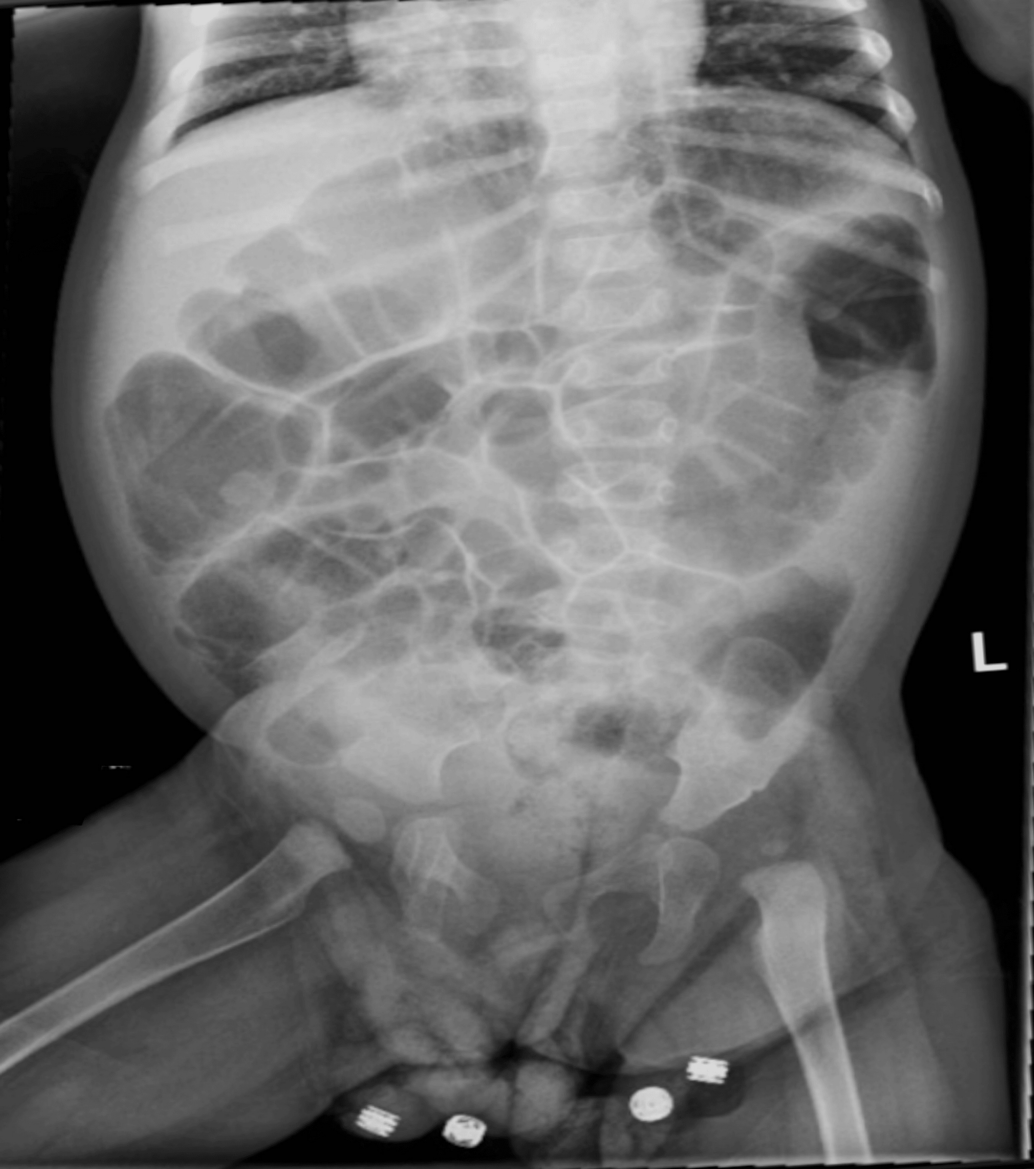
The patient’s abdominal X-ray on her second day of hospitalization revealed no evidence of obstruction, organomegaly, or mass effect. Normal gas pattern throughout. No dilated loops to suggest obstruction. No air-fluid level.

On physical examination, abdominal distension was noted. A plain abdominal radiograph revealed diffuse bowel gas (Figure 2). The patient was on a regular pediatric diet, was having regular bowel movements, and did not display signs of obstruction. Given the absence of obstructive symptoms and normal bowel function, the distension was attributed to aerophagia. A therapeutic trial of simethicone and a glycerin suppository led to the resolution of abdominal distension.

**Figure 3:**
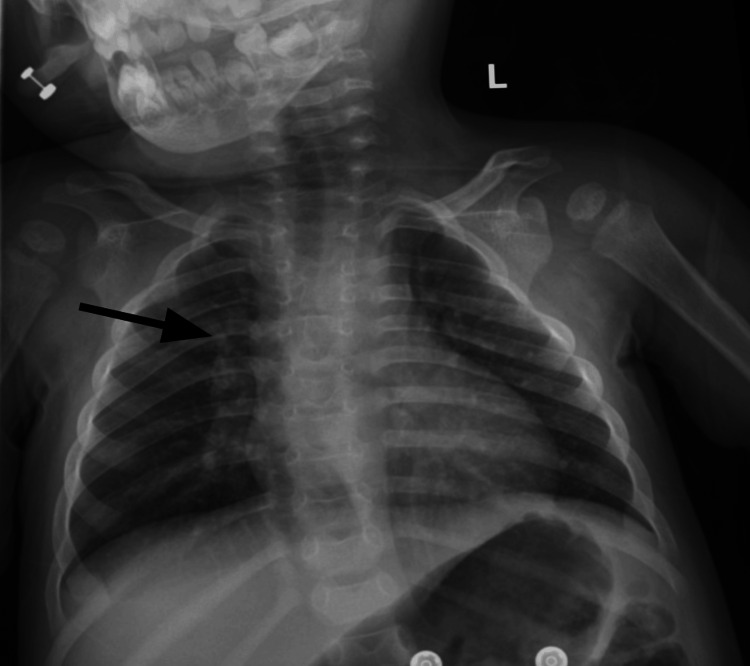
The patient’s chest X-ray on her third day of hospitalization demonstrated perihilar and peribronchial thickening, again consistent with bronchiolitis (black arrow).

On the third day of admission, a trial of fluticasone propionate was given to relieve the upper airway congestion. Another repeat chest X-ray and soft tissue neck X-ray were performed, showing perihilar peribronchial thickening without signs of lobar consolidation, pleural effusions, enlarged epiglottis, pharyngeal distention, or airway steepling (Figure 3). Of note, her congestion persisted, and rhonchi and rales were heard in the upper lobes; however, the patient remained afebrile.

On the fourth day of admission, the patient's croup had resolved, with decreased stridor and reduced wheezing. Her otitis media also showed clinical improvement, with the resolution of tympanic membrane erythema and bulging. She was also tolerating oral intake with adequate urine output and had an oxygen saturation of 95-100% on room air. The patient's initial Westley Croup score was 5, reflecting moderate croup. By the time of discharge, her score had improved to 3, indicating mild croup and a favorable clinical response.

Additionally, throughout her hospitalization, she was noticed to have brief staring spells, lasting up to two minutes. These events were observed by the care team and discussed with neurology. She was carefully monitored with frequent vital signs and neurological exams. No EEG monitoring was performed. To reduce the risk of developing seizures, her at-home seizure medications were continued, along with lorazepam and intranasal midazolam for severe seizure breakthroughs. Although suggestive of seizure-like activity, the staring spells were infrequent, and she did not develop any clonic or tonic activity. The patient remained alert and responsive afterward without signs of post-ictal confusion or drowsiness.

Her prognosis was discussed with her caregivers, and the patient was scheduled to follow up with her primary medical doctor in 2-3 days. The family understood that the patient was to continue the prednisone and azithromycin for two more days for a total of five days, albuterol every four hours as needed, budesonide every 12 hours for a total of 14 days, and to continue at-home seizure medications (Table 2).

**Table 2:** Patient’s drug regimen.

Drug	Dosage	Route	Frequency	Duration in hospital (days given)
Racepinephrine(Asthmanefrin) 2.25 % nebulizer solution 11.25 mg	0.5 mL	Nebulized	Every 2 hours	2 days
Albuterol (2.5 mg/3 mL nebulizer solution)	3 mL (2.5 mg total)	Nebulized	Every 4 hours as needed	4 days
Azithromycin (Zithromax) 200 mg/5 mL suspension	2.6 mL (104 mg total)	Oral	Once daily	3 days
Budesonide (Pulmicort) 0.5 mg/2 mL nebulizer solution	2 mL (0.5 mg total)	Nebulized	Twice daily (morning and evening)	4 days
Prednisolone (Orapred) 15 mg/5 mL solution	3.5 mL (10.5 mg total)	Oral	Every 12 hours	2 days
Clobazam (Onfi) 2.5 mg/mL suspension	2 mL (5 mg total)	Oral	Twice daily	4 days
Levetiracetam (Keppra) 100 mg/mL solution	2.5 mL (250 mg total)	Oral	Once in the morning and once before bedtime	4 days
Oxcarbazepine (Trileptal) 300 mg/5 mL suspension	2.5 mL (150 mg total)	Oral	Once in the morning and once before bedtime	4 days

## Discussion

This case highlights the important implications of managing respiratory disease in pediatric patients with complex comorbidities. This patient was initially admitted to the hospital with symptoms of croup and was successfully treated with racemic epinephrine and steroids. For severe stridor with sternal retractions, irritability, and distress, the initial step was to optimize comfort by placing the child on the parent’s lap and supplying oxygen. Next, after nebulized epinephrine and oral dexamethasone, patients like the one presented in this study who responded poorly should be administered a repeat dose of nebulized epinephrine or undergo further evaluation by the pediatric intensive care unit. If the response is favorable, patients should be observed for two hours and discharged home if symptoms resolve. Hospital admission should be considered if the patient has moderate respiratory distress, stridor at rest, or sternal retractions both with and without agitation and weakness [7].

Further investigation revealed that this patient had mycoplasma pneumonia and otitis media, which were subsequently treated with azithromycin and ceftriaxone. In one study, which grouped *Mycoplasma pneumoniae* and *Chlamydia pneumoniae* by polymerase chain reaction in association with both acute lower and upper respiratory tract infections, children who were treated with azithromycin demonstrated 100% resolution of symptoms at one month compared to 77% of those who were not treated [8].

Additionally, likely due to oxygen deprivation, metabolic changes, and systemic inflammation caused by the acute infection, this patient experienced seizure-like staring episodes [9]. Sudden unexpected infant deaths are commonly caused by seizures, which can be induced by hypoxia and apnea in younger infants and go undetected if they are brief or lack obvious symptoms, making early diagnosis difficult and timely intervention critical [10]. Continuous monitoring of vital signs and oxygen saturation was essential throughout this patient’s hospitalization. Moreover, findings have shown that certain respiratory viruses, such as human rhinovirus, enterovirus, influenza, coronavirus, adenovirus, and parainfluenza, are more strongly associated with febrile seizures than other respiratory pathogens [11]. This study also found that factors such as sex, fever duration, family history of seizures, the type of virus involved, and the results from lumbar punctures were important considerations when diagnosing and treating children with seizures effectively [11]. Respiratory distress and certain viruses can lower the seizure threshold in children. In particular, the upregulation of proinflammatory cytokines, interleukin-1 beta (IL-1β), and cyclooxygenase-2 (COX-2) leads to the breakdown of the blood-brain barrier [12]. This initial reaction sets off a cascade of events, including the production of transforming growth factor-beta (TGF-β) and nuclear factor kappa-light-chain-enhancer of activated B cells (NFkB) in microglia, astrocytes, and neurons that can trigger an inflammatory response and neuronal excitability that alter the seizure threshold [12].

Future research should examine how medications can be adjusted in patients with complex medical histories who develop respiratory issues, both during and after the acute phase. Additionally, this case examines the potential link between the patient's respiratory illness and the subsequent delayed onset of seizures after admission. A multidisciplinary approach provided comprehensive care via reassurance, meticulous examination, and collaborative effort in the context of acute illness.

## Conclusions

This case illustrates the crucial role of racemic epinephrine, steroids, antibiotics, and seizure management protocols for this pediatric patient with a significant complex medical background. Research on chromosome 16p13.3 microduplication remains limited, with few resources available on how to combat respiratory illnesses, as well as manage seizures during and after an acute episode. In this case, the patient’s croup, pneumonia, and otitis media were effectively treated with the appropriate medications, but the new emergence of staring spells indicates a potential interaction between respiratory infections and seizures, although the exact pathophysiological link between the two remains unclear. Clinicians should be aware of the possibility that acute illness may exacerbate seizures in this subset of individuals. As demonstrated in this case, maintaining the patient’s at-home seizure medications during the acute illness proved to be beneficial, and showed no breakthrough seizures, medication intolerance, or drug-drug interactions.

The patient’s croup was managed with systemic and inhaled corticosteroids, racemic epinephrine, bronchodilators, and suctioning. Mycoplasma infection was treated with azithromycin, while acute otitis media was managed with ceftriaxone. Her underlying seizure disorder was closely monitored under neurology, with the continuation of her home antiepileptic medications. The patient’s treatment was carefully individualized, resulting in gradual respiratory improvement and resolution of acute symptoms by hospital day 4. Given the uniqueness of each individual and case, adjustments to seizure management may be necessary, prompting further research in these high-risk populations to better understand how to ensure more effective treatment in a timely manner.
